# 40-Hz Blue Light Changes Hippocampal Activation and Functional Connectivity Underlying Recognition Memory

**DOI:** 10.3389/fnhum.2021.739333

**Published:** 2021-12-16

**Authors:** Zhenglong Lin, Gangqiang Hou, Youli Yao, Zhifeng Zhou, Feiqi Zhu, Linjing Liu, Lingwu Zeng, Yatao Yang, Junxian Ma

**Affiliations:** ^1^College of Electronics and Information Engineering, Shenzhen University, Shenzhen, China; ^2^Department of Radiology, Shenzhen Kangning Hospital, Shenzhen Mental Health Center, Shenzhen, China; ^3^Department of Physiology, School of Basic Medical Sciences, Shenzhen University Health Sciences Center, Shenzhen University, Shenzhen, China; ^4^Cognitive Impairment Ward of Neurology Department, The Third Affiliated Hospital of Shenzhen University Medical College, Shenzhen, China

**Keywords:** blue light, fMRI, hippocampus, memory, functional connectivity, frequency

## Abstract

Research on light modulation has typically examined the wavelength, intensity, and exposure time of light, and measured rhythm, sleep, and cognitive ability to evaluate the regulatory effects of light variables on physiological and cognitive functions. Although the frequency of light is one of the main dimensions of light, few studies have attempted to manipulate it to test the effect on brain activation and performance. Recently, 40-Hz light stimulation has been proven to significantly alleviate deficits in gamma oscillation of the hippocampus caused by Alzheimer’s disease. Although this oscillation is one of the key functional characteristics of performing memory tasks in healthy people, there is no evidence that 40-Hz blue light exposure can effectively regulate brain activities related to complex cognitive tasks. In the current study, we examined the difference in the effects of 40-Hz light or 0-Hz light exposure on brain activation and functional connectivity during a recognition memory task. Through joint augmentation of visual area activation, 40-Hz light enhanced brain areas mostly in the limbic system that are related to memory, such as the hippocampus and thalamus. Conversely, 0-Hz light enhanced brain areas mostly in the prefrontal cortex. Additionally, functional connection analysis, with the hippocampus as the seed point, showed that 40-Hz light enhanced connection with the superior parietal lobe and reduced the connection with the default network. These results indicate that light at a frequency of 40 Hz can change the activity and functional connection of memory-related core brain areas. They also indicate that in the use of light to regulate cognitive functions, its frequency characteristics merit attention.

## Introduction

Light is essential for vision, which is the principal sensory channel for humans to perceive external information and has a crucial impact on human physiological functions (Vandewalle et al., 2009; LeGates et al., 2014). Generally, abnormal light environments (e.g., shortening daylight in winter or exposure to light during a sleep stage) can cause problems in many areas, such as circadian rhythm, sleep, mood, and cognitive function (Altimus et al., 2008; Zelinski et al., 2014; An et al., 2020). Given how sensitive the human brain is to light, a growing number of studies have attempted to utilize the light field as a tool to modulate the brain so as to improve cognition and behavior and prevent and improve mental illness (Glickman et al., 2006; Huang et al., 2019, 2021; Killgore et al., 2020b). Correspondingly, based on the brain’s response to the input light, it is important to modulate the light field and to select specific light parameters for different cognitive functions to optimize the light regulation effect.

The physiological adjustment of light is usually called non-visual because it has characteristics that can be distinguished from the visual system (Foster, 2005; Vandewalle et al., 2009). Unlike the retinal system, which mediates visual information, the physiological effect of light regulation has been shown to occur even in completely blind people and rodents (LeGates et al., 2014). Thus, this effect of light is non-visual. Such a non-visual effect of light is received by intrinsically photosensitive retinal ganglion cells (ipRGCs) in the retina and projected to the suprachiasmatic nucleus (SCN) and the pineal gland to regulate biological rhythms, melatonin secretion, and sleep; it has also exhibited performance enhancement effects in visual search, digit recall, and logical reasoning tasks (Chou et al., 2002; Dacey et al., 2005; Guler et al., 2008). Neurons respond to different wavelengths of light, and this response has been found to be associated with changes in cognitive function (Vandewalle et al., 2009; Park et al., 2020). A large amount of evidence has shown that ipRGCs are most sensitive to the blue wavelength with regard to non-visual effects (Berson et al., 2002). Blue light (460-nm wavelength) has not only shown a stronger regulatory effect than green light (550-nm wavelength) in working memory tasks but has also shown broader induction of brain activity than violet light (430-nm wavelength) (Vandewalle et al., 2007a,b). In addition, studies have indicated that the non-visual effects of light are also directly or indirectly projected through ipRGC and SCN to the peripheral hypothalamus, thalamus, striatum, brainstem, and limbic structures (Aston-Jones et al., 2001; Luo and Aston-Jones, 2009). These projections not only regulate arousal and alertness, which are physiological states that support cognitive processes, but also regulate higher-order cognitive functions (Drevets, 2001; Aston-Jones and Cohen, 2005; Russo and Nestler, 2013; Ferreira and Castellano, 2019). For example, research has indicated that blue light is superior to other wavelengths of light in regulating drowsiness and improving alertness by increasing brain functional connectivity and reducing task-related neural resource requirements (Alkozei et al., 2016; Killgore et al., 2020a). Furthermore, blue light has elicited priority responses in areas of the brain that relate to emotional processes (Vandewalle et al., 2010).

The use of light as a regulator to intervene in behavior and higher-order cognitive functions has varying effects owing to the diversity of its variables, the response characteristics of neurons, and the cognitive-specific functional network. There is also evidence that light-regulating effects are related to intensity and exposure time. While the limbic system typically responds quickly to light stimulation, higher cortical areas respond at a slower rate, requiring stronger intensity and a longer duration of exposure. As such, studies have indicated that the performance-enhancing effects of light exposure are significantly observable after 30 min of exposure, while other studies with less exposure time (e.g., below 21 min) did not find a similar enhancement (Vandewalle et al., 2009; Alkozei et al., 2016). Furthermore, longer exposure times and higher exposure intensities seem to cause differences in the degree of changes in brain activity (Vandewalle et al., 2006), and such effects can be maintained longer (Alkozei et al., 2016; Killgore et al., 2020a).

These findings indicate that the manipulation of different dimensions of light can significantly optimize the benefits of interventions. Note that the light sources used in these studies are all 0 Hz (natural light, simulated natural light and LED light), and none of them flicker. Taking this into account, the manipulation of frequency, which is a major dimension of light, may also play a comparable role in light interventions. Different types of neurons in the brain interact to form unique rhythmic oscillations (Buzsaki, 2006; Buzsaki and Wang, 2012). These oscillations have been shown to be crucial components in several cognitive functions, including memory (Buschman et al., 2012; Salazar et al., 2012). For example, MEG research has indicated that in working memory tasks, gamma power increases with the increase in memory items (Howard et al., 2003). Other research has indicated that the hippocampus, the most critical brain area for memory, is correlated with higher gamma intensity in monkeys and humans (Lisman and Jensen, 2013). These neuronal oscillations can be awakened synchronously by external stimuli. A functional magnetic resonance imaging (fMRI) study measured the real-time response of the brain to flickering visual stimuli with a frequency varying from 1 to 40 Hz, and revealed that visual stimuli could selectively activate cortical areas and regulate specific brain connections (Chai et al., 2019). In another ERP study, it was verified that 40, 60, and 80-Hz light stimulation induced the human brain response, and the greatest amplitude was always found in the primary visual cortex (Jones et al., 2019). However, these studies did not show changes in the hippocampus or determine whether there were changes in brain activity related to memory tasks. Different cognitive functions have unique neural oscillations characteristics. For example, attentional-related functions are related to delta band oscillations and memory functions to gamma oscillations. Thus, in terms of regulating memory function, selective use of light frequency (e.g., 40 Hz) to modulate the gamma oscillation frequency closely related to specific memory function may be one way to maximize the benefits of light intervention.

The value of this work is verification of the effect of light regulation on the cognitive regulation of memory in healthy people. It can also be used as a reference for light-mediated neuromodulation for other cognitive functions, including improving attention and learning efficiency, maintaining cognitive ability, and slowing down degeneration. It may even provide some clues for follow-up human clinical research. Relevant clinical research has been mostly limited to animal experiments. Animal studies have indicated that flashing blue light with a frequency of 40 Hz can significantly improve circadian rhythm disorders in mice with Alzheimer’s disease (APP/PS1), which further verifies the effect of light regulation on frequency characteristics (Yao et al., 2020). In addition, neurological disease appears deficient in the gamma band of neural oscillation, and such deficits can be compensated by exposure to external sensory stimulation (Uhlhaas and Singer, 2006; McDermott et al., 2018). Aiming at abnormal gamma oscillation in the Alzheimer’s disease (AD) mouse model, Tsai et al. used light stimulation to affect gamma oscillation of the visual area, significantly increasing the activity of microglia, reducing the precipitation of amyloid beta (Aβ) protein, and ultimately providing a wide range of effects in the whole brain to improve behavior and pathological outcomes (Iaccarino et al., 2016). Another study showed that using gamma frequency light to interfere with stroke model mice with impaired memory not only regulated gamma oscillation in the hippocampus but also enhanced the strength of nerve synapses, improved neuroplasticity, and promoted the recovery of memory function (Yao et al., 2020). Note that these studies showed that the oscillating effect induced by flickering light had a significant frequency preference. Specifically, light presented at 40-Hz frequency showed a stronger regulatory effect than other frequency bands and a higher association with memory-related brain areas, such as the hippocampus. Meanwhile, this modulation of visual areas typically has a significant effect on the improvement of gamma oscillations, microglia activity, and synaptic plasticity (Iaccarino et al., 2016; Zheng et al., 2020). Considering that microglia mediate synaptic pruning, which is a normal part of brain development (Hong et al., 2016a,b) and plays an important role of gamma oscillation in memory function, it is reasonable to speculate that the regulatory benefits of 40-Hz light also affect neurons and related memory functions in healthy brains. However, few studies have explored the effect of 40-Hz flashing blue light exposure on the regulation of brain activity patterns related to high-level cognitive tasks in healthy brains. In addition, although the role of 0-Hz light in improving arousal and alertness has been verified, its exact kind of promotive effect in terms of memory and cognitive function has not been determined, nor has it been determined whether this effect is equivalent to the effects observed at 40 Hz.

In the current study, we attempted to examine the effect of 40 and 0-Hz light exposure on the regulation of brain activity patterns in healthy people when performing memory tasks. We utilized fMRI to measure both brain activity and changes in functional connectivity during the performance of a modified recognition memory task under 40 and 0-Hz light intervention. We hypothesized that these interventions would lead to distinct patterns of activity during a recognition memory task. Furthermore, we hypothesized that 40-Hz light exposure would be more closely associated with the regulation of core areas of the memory-related network compared with exposure to 0-Hz light.

## Materials and Methods

### Participants

Forty-four (12 female, 32 male) volunteers (aged 18–24 years; mean = 20.3, *SD* = 1.58) were recruited from Shenzhen University to participate in the study. The list of participants was confirmed 2 weeks before the start of the experiment. These participants were randomly divided into two groups that received one of two stimulation protocols: 1 h of 40-Hz or 1 h of 0-Hz light exposure in the interval between the two memory experiments. All the participants were right-handed, with normal, or corrected to normal, vision and hearing, and free of head injury, psychiatric problems, neurological impairment, or language impairment. Written informed consent was obtained from all subjects before the scanning session.

### Light Exposure Device

**FIGURE 1:**
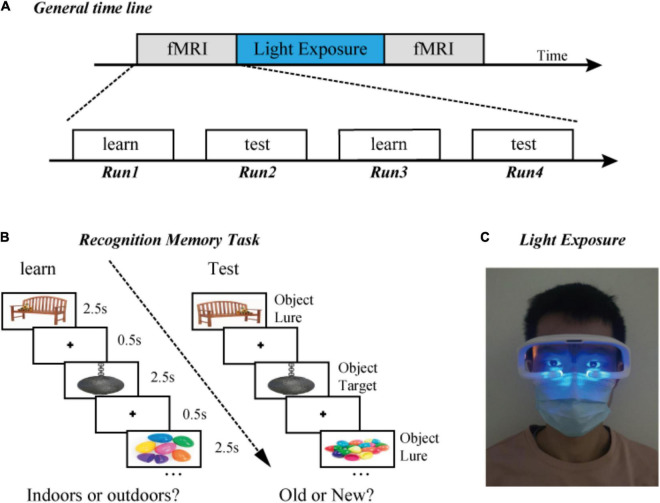
Experimental design. **(A)** General timeline. **(B)** Examples of the presentation format in the “indoors/outdoors” and “old/new” judgment task. Participants learned a series of objects, which was followed by a recognition test. **(C)** Diagram of light exposure. All participants received 0 or 40-Hz blue light stimulation in a dark room for 1 h.

After the first fMRI experiment, each participant received 0 or 40-Hz blue light stimulation in a dark room. The blue light-emitting diode (LED) device used in this experiment was sourced from commercial glasses made by Shenzhen Wyselife Co., Ltd. Two LEDs (centroid wavelength = 472 nm, time-frequency modulation frequency = 0 or 40 Hz, irradiation power density = 0.03 mW/cm^2^) were installed on the lower edge of the left and right frames of the glasses (Figure 1C). The maximum luminous axis of each LED is generally vertical to the plane of the frame, and the central point of illumination is below the eye. At the same time, the illumination range can cover the upper side of the eye (see the patent specification for details, Patent number ZL201830101531.9, China).

### Experimental Procedure

Participants came to the laboratory 1 week before the day of the experiment to undergo introduction and practice for the experimental process. In order to prevent interference from the learning effect, the stimulus materials used for practice were different from those used in the formal experiments. The effects of neuromodulation may be influenced by individual differences, and inherent cognitive ability may be one of the factors that affect the regulation effect. In order to determine whether working memory ability is correlated with the degree of change after being treated with 40 and 0-Hz light, the digit span and 2-back tasks were performed on the same day. The digit span uses the Wechsler Adult Intelligence Scale (WAIS-III) (Wechsler, 1997), and the total raw score for the digit span is the sum of both of forward and backward test. In the current study, a 5-min custom designed 2-back task was used, referring to a widely used visual task (Alkozei et al., 2016). Participants were visually presented with a sequence of numbers and required to judge whether the current number matched the number from two steps earlier by pressing a button under the index finger for “yes” or a button under the middle finger for “no.” During the task, accuracy and response time were recorded.

Participants were required to complete all three parts of the experiment (Figure 1A). First, in an fMRI experiment, participants completed four runs of learning and testing, six blocks per run. Second, after the first scan, the participants left the MRI room and were exposed to optical stimulation in a dark room for 1 h. Human research has indicated that the neurons’ response to the gamma band has the largest amplitude at 40 Hz. Considering that the MRI scanning time had been optimized, only 40 and 0 Hz were selected for current experiment rather than other frequencies. Third, in a subsequent fMRI experiment, participants repeated four runs of the learning and test, with novel stimuli (Figure 1B).

The experiment included a learning phase followed by a testing phase. The participants were required to classify a series of 60 objects as “Indoors” or “Outdoors” during the learning phase. In the testing phase, participants were required to classify a series of 60 objects as “Old” or “New.” During the testing phase, 15 objects were identical to learned objects, and 45 objects were perceptually similar lures. Based on the prior similarity verified by previous research (Lacy et al., 2011; Stark et al., 2013), lure objects were chosen from databases in the similarity range of 2–4 (out of the full range of 1–5).

The task was a modified version one from a previous study (Reagh et al., 2018; Klippenstein et al., 2020). We optimized it for the current study. The stimuli were presented using a block design, with six blocks per run. Both learning and testing runs included five 16-s rest periods between object blocks. Each object was displayed for 2.5 s, with a 0.5-s inter-stimulus interval within each 30-s block. Sixty learning objects were displayed sequentially, followed by 60 test trials, equaling one set (two runs). Participants had to perform two sets (four runs) in the experiment before and after light exposure, respectively. During the scan, accuracy and response time were recorded *via* a button press. All participants were required to respond as quickly and accurately as possible. The stimulus sequence was completely random and unique to each block.

### Functional Magnetic Resonance Imaging Data Acquisition

During fMRI scanning, the participants lay in a supine position in the MRI tunnel while looking at the stimulus on the screen in the mirror, which was fitted to the head coil (viewing angle, 5 × 2.5°). Scanning was performed at Kangning Hospital in Shenzhen, using a 3T GE 750 MRI scanner with an 8-channel head coil. A gradient–echo planar imaging (EPI) sequence was used [TR (repetition time)/TE (echo time) = 2000/30 ms; flip angle = 90°; voxel size of 3 × 3 × 3.5, with a 0-mm gap]. After functional scanning, an anatomical image was acquired using a T1-weighted spin echo pulse sequence with a 1-mm^3^ isotropic voxel size.

### Data Analysis

The imaging data were analyzed using the Statistical Parametric Mapping package (SPM8; Wellcome Department of Cognitive Neurology, London, United Kingdom) and MATLAB 7.5 (MathWorks, United States). The first six volumes of each task were excluded from the analysis to eliminate the non-equilibrium effects of magnetization. Scans were spatially realigned to the first volume of the first time series. The T1-weighted anatomical images were co-registered to the first scan of the functional image and subsequently normalized to the standard T1 template image, according to the Montreal Neurological Institute (Cocosco et al., 1997). The data were spatially smoothed with an isotropic 6-mm full-wide half-maximum Gaussian kernel. A general linear model (GLM) was fitted to the fMRI data for each subject (Friston et al., 1998). The blood-oxygen-level dependent (BOLD) signal for all tasks was modeled using boxcar functions that were convolved using the canonical hemodynamic response function. At the first level, one-sample *t*-test analysis was conducted. All four runs (two learnings and two tests per run) were included in a design matrix for each subject and rested between blocks as the baseline for estimating the task condition. Subsequently, we evaluated the linear contrasts in each subject and obtained contrast images for the random-effect group analysis. A second-level analysis was performed with a full factorial design to obtain each group result and group comparisons. The SPM [T] maps were generated at a threshold of *p* < 0.05 and were corrected with a false discovery rate (FDR) in the whole brain.

### Functional Connectivity Analysis

We also examined seed-to-voxel functional connectivity using the CONN-fMRI toolbox (Whitfield-Gabrieli and Nieto-Castanon, 2012) for SPM8 (Wellcome Department of Cognitive Neurology, London, United Kingdom). ROIs, which were defined based on the parts of the brain showing significant differences in the signal before and after optical stimulation, were used as seeds for functional connectivity analyses. The CompCor strategy implemented in CONN-fMRI was used to reduce the effect of nuisance covariates, such as (1) fluctuations in the BOLD signal from CSF, (2) realignment parameter noises, (3) white matter and its derivatives, and (4) task effects and their first temporal derivatives. Band-pass filters (0.008 Hz < *f* < infinite) were used to filter data (Yang et al., 2018). At the first level, Pearson’s correlation coefficients between the time course of each ROI and all other voxels were computed in each condition separately. Data from before and after light exposure were transformed into Fisher’s *Z*-scores. At the group level, frequency differences in functional connectivity during the two kinds of light conditions (i.e., 40 vs. 0 Hz) were examined using a two-way ANOVA with an uncorrected voxel-wise threshold of *P* < 0.001 and an FDR-corrected cluster-size threshold of *P* < 0.05. To test the reliability of differences across frequency and light exposure while accounting for the possible violation of general linear model assumptions, non-parametric permutation tests, with 1,000 permutations, were performed to confirm the results of parametric tests using an uncorrected voxel-wise threshold, with *P* < 0.001 and an FDR-corrected cluster-mass threshold of *P* < 0.05 (Nichols and Holmes, 2002). Correlation analysis was conducted to investigate the relationship between functional connectivity and working memory (behavior scores of the digit span test and the 2-back task).

## Results

### Behavioral and Imaging Results

**FIGURE 2:**
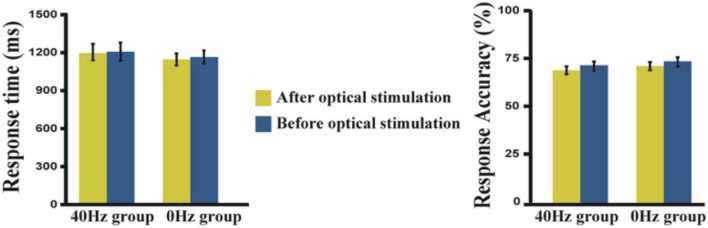
Response time and accuracy in the 40-Hz group and 0-Hz group. A two-way ANOVA result shows that neither the main effect nor an interaction can be observed. Yellow bar, after light exposure; Blue bar, before light exposure. The error bars show the standard deviation of the mean.

The results show reduced response times and lower accuracies after optical stimulation when compared with data acquired before optical stimulation (Figure 2). However, these differences are not statistically significant. We performed a two-way ANOVA (before and after optical stimulation as the within-subjects factor and participants in the two groups as the between-subjects factor) for the response time, and neither the main effect nor an interaction was observed. In terms of the results of the 2-back task performed before the experiment, the two groups showed no difference in reaction time [*t*(42) = −1.124, *p* = 0.902] or accuracy [*t*(42) = −1.001, *p* = 0.323]. Likewise, there is no difference in the scores of the two groups in the digit span test [*t*(42) = −1.533, *p* = 0.133].

**FIGURE 3:**
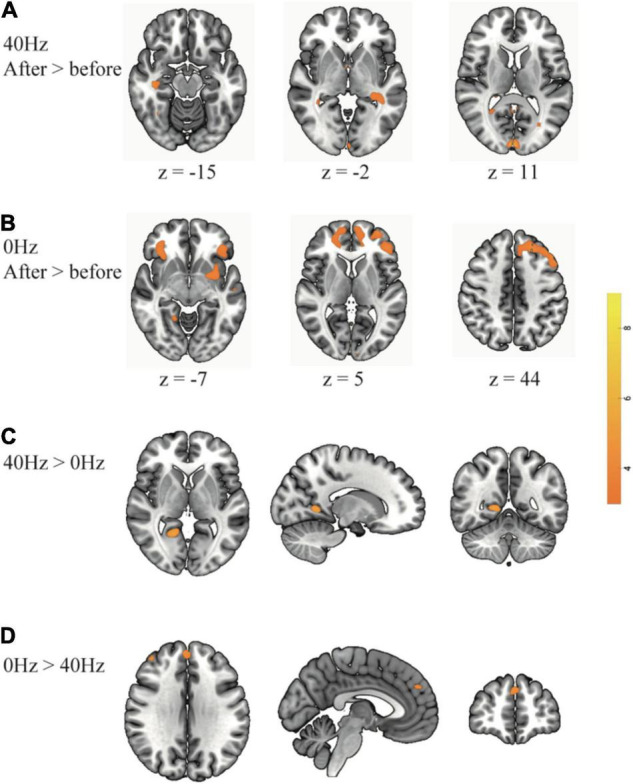
Greater brain activation is associated with comparisons between before and after optical stimulation in each group: **(A)** The 40-Hz group shows additional activation in the bilateral hippocampus and visual cortex. **(B)** The 0-Hz group shows increased activation in the prefrontal cortex. For a direct comparison between two groups: **(C)** The 40-Hz group shows greater activation in the left para-hippocampus and **(D)** The 0-Hz group shows greater activation in the middle frontal cortex and medial frontal cortex.

**FIGURE 4:**
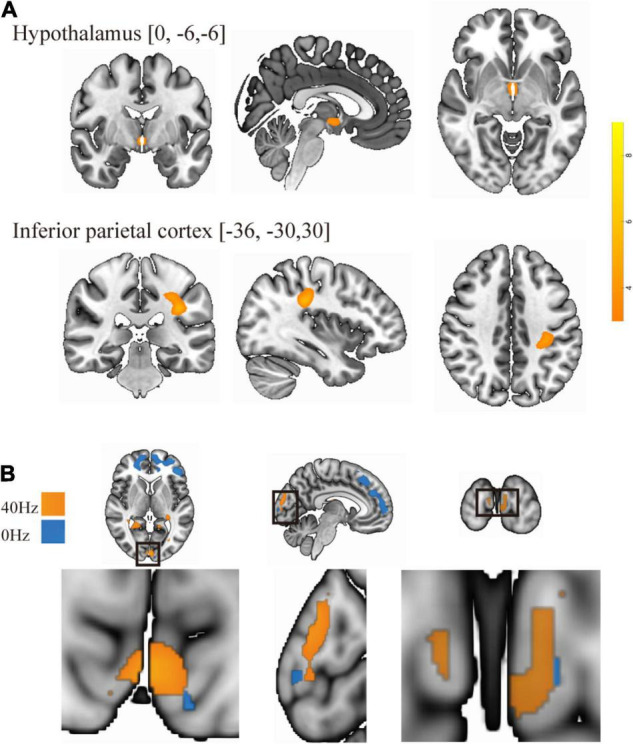
**(A)** There is a significant interaction of before and after stimulation × light condition in the inferior parietal cortex and thalamus/hypothalamus. **(B)** Overlay of group differences in enhanced brain activity to frequency: the 40-Hz group is in yellow, and the 0-Hz group is in blue.

As shown in Figure 3A, only the 40-Hz group shows additional activation in the bilateral hippocampus region during the object discrimination task after optical stimulation. Conversely, the 0-Hz group shows additional activation in the prefrontal cortex (PFC), which is known for object discrimination, after optical stimulation (Figure 3B). For a direct comparison between two groups after optical stimulation, only the 40-Hz group shows greater activation in the left para-hippocampus (Figure 3C). In contrast, individuals in the 0-Hz group show greater activation in the left middle frontal gyrus and left medial frontal gyrus when compared with the 40-Hz group (Figure 3D). However, no regions are activated more strongly in the reverse comparison. Additionally, a significant interaction of before and after stimulation x stimulation condition can be seen in the inferior parietal cortex and thalamus/hypothalamus (Figure 4A). These results are summarized in Table 1.

**TABLE 1:** Intra- and inter-group differences under the condition of light stimulation (*P* < 0.05, FDR corrected with a minimum cluster extent of 10 contiguous voxels).

Contrast	Anatomical region	MNI coordinate			*T* value	Voxels
		x	y	z		
40-Hz group After > Before	L Inferior Parietal Gyrus	−36	−30	30	6.16	103
	L Hippocampus	−30	−33	0	4.08	27
	L Occipital Gyrus	−15	−102	−4	3.67	10
	R Occipital Gyrus	3	−93	9	5.75	131
	R Hippocampus	39	−21	−18	4.28	11
0-Hz group After > Before	L Superior Frontal Gyrus	−18	24	60	5.56	403
	L Middle Frontal Gyrus	−39	6	60	5.10	24
	R Inferior Frontal Gyrus	63	15	24	4.62	40
	L Middle Frontal Gyrus	−33	57	6	4.45	272
	L Inferior Frontal Gyrus	−39	36	3	4.41	105
	R Inferior Frontal Gyrus	36	33	−9	4.14	342
	R Post-central Gyrus	42	−18	27	4.07	29
	R Middle Frontal Gyrus	45	30	42	3.58	14
	L Superior Temporal Gyrus	−57	−18	−6	3.42	11
	L Middle Temporal Gyrus	−54	−33	0	3.34	15
After 40 > 0-Hz	R Parahippocampus Gyrus	21	−51	3	5.18	40
	Cingulate Gyrus	0	−18	27	4.50	16
After 0 > 40-Hz	L Medial Frontal Gyrus	−12	39	30	4.84	28
	L Middle Frontal Gyrus	−30	27	27	4.42	13
Interaction	L Inferior Parietal Gyrus	−36	−30	30	5.75	39
	Hypothalamus	0	−6	−6	4.48	5

*L = left and R = right.*

### Functional Connectivity Results

**FIGURE 5:**
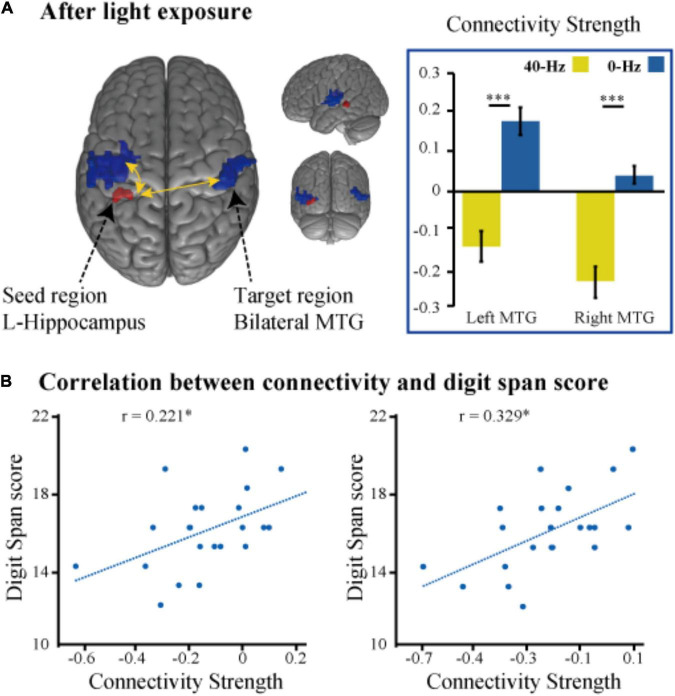
**(A)** Intergroup differences in functional connectivity of the left hippocampus seed during the memory judgment task after the optical stimulation. **(B)** Scatter plots of the correlation between functional connectivity strength (hippocampus with the bilateral MTG) and score of the digital span test. *P < 0.05, ^***^P < 0.001.

**FIGURE 6:**
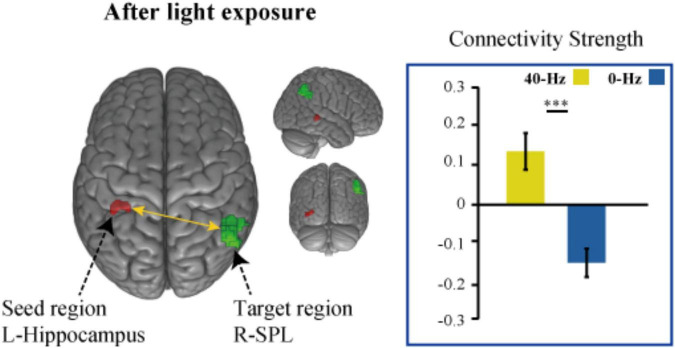
Intergroup differences in light-exposure-induced strength of functional connectivity between the left hippocampus and SPL. The functional connection between hippocampus and SPL enhanced after 40-Hz light exposure but not 0-Hz light exposure. ^***^P < 0.001.

To identify optical stimulation effects and stimulation condition-related regions, we also examined functional connectivity to specifically reveal the difference in connection strength in the entire brain between the two groups during the object discrimination task. The bilateral hippocampi, inferior parietal lobule, and thalamus areas, which were activated as a result of the object discrimination task, were recruited as a seed to perform seed-to-voxel functional connectivity analyses. Functional connectivity from the left hippocampus to right angular gyrus and bilateral middle temporal gyrus was obtained. The 40-Hz blue light exposure group exhibits weaker functional connectivity between the hippocampus and bilateral middle temporal gyrus (MTG) after light exposure conditions than the 0-Hz blue light exposure group, which reveals connectivity suppression [*t*(42) = −6.099, *P* < 0.001 and *t*(42) = −5.902, *P* < 0.001] (Figure 5A). The connectivity strength between the hippocampus with the bilateral MTG is positively correlated with score of Digit Span test [*r* = 0.221, *P* = 0.027 and *r* = 0.329, *P* = 0.005] (Figure 5B). Meanwhile, we can see significant inter-group differences in light-exposure-induced functional connectivity between the left hippocampus and SPL [*t*(42) = 4.843, *P* < 0.001], which are driven by reduced functional connectivity in the 0-Hz blue light exposure group but increase functional connectivity in the 40-Hz blue light exposure group during the task (Figure 6). In contrast to the 40-Hz group, no positive functional connectivity is observed between the hippocampus and MTG during memory retrieval processing after 0-Hz light exposure.

## Discussion

The goal of the present study was to investigate the changes and differences in the regulation of brain activity patterns in healthy people after 40 and 0-Hz light regulation when performing memory tasks. We also performed functional connectivity analysis related to memory tasks in order to observe changes in network connectivity patterns throughout the brain and further investigate relationships between neural activity, behavioral performance, and cognitive ability. The behavioral results show that performance was not influenced by light. Changes in neural activity without corresponding changes in cognitive performance were noted. This is similar to results presented by many previous neuroimaging studies and may have stemmed from the ceiling effect of healthy young people; that is, the degree to which cognitive performance can be improved is relatively limited (Tort et al., 2009). It is possible that improvements in results require longer, repeated, or a higher intensity of light exposure (Phipps-Nelson et al., 2003). In addition, it is possible that the improvement of cognitive performance is related to the kind of task that is performed. For example, under the same non-flickering blue light for 30 min of exposure, the results of the study that used the 2-back task reflect an improved cognitive performance, while the results of the study using the Multi-Source Interference task reflect no change in cognitive performance (Alkozei et al., 2016; Killgore et al., 2020a). The difference in brain activity patterns when performing specific cognitive tasks may be one of the factors affecting whether light exposure can effectively affect cognitive performance.

Previous studies have indicated that visually induced gamma oscillation is not limited to the visual cortex (Pastor et al., 2003; Adaikkan et al., 2019). Our results also show that the activity of the visual area was enhanced after both 40 and 0-Hz light stimulation. However, the intensity of the enhancement differed between the two groups (Figure 4B). Additional memory-related brain-related areas showed enhanced activation, with the frequency-induced areas being distinct. The changes in the hippocampus and other memory-related brain areas were primarily observed in the 40-Hz group, while the areas of 0-Hz enhancement were mostly observed in the PFC. Furthermore, functional connection changes of memory-related brain networks were only observed in the 40-Hz group, especially when the left hippocampus was used as a seed point. This is important because the degree of functional connectivity change shows a moderate correlation with the score of the digit span test, which was performed before the experiment.

Compared with the 0-Hz light, the amplitude and range of the activity enhancement in the visual area induced by 40-Hz light were larger. Additionally, an increase in activation of the hippocampus was only observed after 40-Hz light stimulation. Our results suggest that 40-Hz light promotes the activation of the hippocampus during memory processing, which is necessary for successful recognition of memory processing (Poppenk et al., 2013; Strange et al., 2014; Hrybouski et al., 2019). Furthermore, these results are consistent with the view that gamma oscillation is closely related to memory functions. Induction of neuronal oscillations through sensory stimulation to regulate memory function has been widely used. In animal models, utilization of one gamma frequency light to improve hippocampal gamma deficits in an effort to treat neurological diseases has been generally confirmed (Barth and Mody, 2011; Verret et al., 2012; Etter et al., 2019). One study examined how induction of gamma oscillations in the visual cortex attenuates cognitive dysfunction in a mouse model of AD (Jones et al., 2019). Similarly, in another study, vertebral neurons in the CA1 region of the hippocampus were effectively protected after mice received low-frequency gamma illumination after two-vessel occlusion surgery (Yao et al., 2020). In addition, transcranial alternating current stimulation-induced gamma oscillation could improve cognitive ability (Florin-Lechner et al., 1996; Banks et al., 2016). The above studies indicated that increased gamma oscillation increases working memory capacity. In order to improve cognitive ability, memory capacity should be increased (Tseng et al., 2016). Note that intrinsic gamma oscillations were not directly measured in this study. However, given that 40-Hz light exposure can effectively improve the excitability of the hippocampus during memory recognition tasks, we speculate that 40-Hz light exposure may be an effective method for entraining gamma oscillations in the hippocampus. A previous study also showed that single exposure to 0-Hz blue light had a subsequent beneficial effect on working memory performance, even after cessation of exposure, and led to temporarily persisting functional brain changes within prefrontal brain regions during a 2-back task (Alkozei et al., 2016). Furthermore, performance in working memory tasks is positively correlated with PFC, indicating that increased prefrontal activation leads to faster decision making. Although the cognitive performance in this experiment was not significant, at least in terms of enhancing PFC activation, 0-Hz light exposure showed an effect similar to this in a previous study. Considering that the memory recognition task in the current study also requires the maintenance of working memory information, as well as subsequent information retrieval that is similar to the n-back task, the results of 0-Hz frequency light-induced enhancement of PFC activation appear to be more reasonable. Combining the results of previous studies and of this experiment, we speculate that for memory-related tasks, especially for tasks that evoke activities in the PFC area, 0-Hz exposure may have a universal effect on the modulation of PFC activities during memory task processing. Indeed, previous studies have indicated that 0-Hz blue light exposure leads to greater activation in the locus coeruleus, which in turn releases norepinephrine throughout the cortex (Strüber et al., 2014; Alkozei et al., 2016). Under the same lighting conditions, given that twice as much 0-Hz light enters the retina as 40-Hz light, this may reflect the increase in the prefrontal lobe BOLD response. We observed this under 0-Hz light conditions but not 40-Hz light conditions. In addition, it is necessary to consider that the participants had been performing the experiment for nearly 2 h before starting the second scan, which may have required more effort in the execution of the recognition task. Previous studies have suggested that fatigue-induced increased activation can occur in PFC (Axmacher et al., 2010; Hilty et al., 2011). In this experiment, the fatigue felt by the participants during the experiment may have been one of the factors that affected the increase of PFC activation.

In terms of brain functional connectivity during recognition memory tasks, the pattern of connectivity between the hippocampus and the SPL is significantly distinct from connectivity between the hippocampus and the MTG. We found that the functional connection between hippocampus and SPL was enhanced after 40-Hz light stimulation. This change usually benefits cognitive functions, such as sensory processing, attention, and memory processing. Decreases in the functional connectivity between cortical areas in patients with mild traumatic brain injury have been observed, but functional connectivity has been found to increase as symptoms improve (van Duinen et al., 2007). Similarly, the functional connection between the SPL and the default mode network in AD patients has also shown improvement (Chao, 2019), with evidence that this symptom improvement is associated with an increase in network connections. The functional connectivity between the hippocampus and early visual areas during memory recognition suggests that cortical-hippocampal interaction supports recognition performance (Paleja et al., 2014). The hippocampus is the core of the memory function network, and other cortices, such as sensory areas, provide additional information to assist, but not directly dominate, memory recognition (Pidgeon and Morcom, 2016). This is consistent with the theory that the cortex, related to visual spatial information processing, does not play a direct role in memory recognition, but provides a visual pattern for the hippocampus to promote memory recall (Rolls, 2018). The 40-Hz entrained gamma oscillations can be used to configure functional neural circuits to selectively transmit sensory information to distributed neural circuits. Furthermore, 40-Hz light may entrain the synchronization of the gamma oscillations in the hippocampus and SPL, and as the excitement period in the oscillation network reaches the peak of excitability, the input will reach peak effectiveness. In view of the fact that SPL is related to the processing of visual spatial information and attention function, the hippocampus-SPL connection change we observed suggests that 40-Hz light enhances the perception of visual spatial information in the process of memory recognition and the transmission of information between regions, which may be beneficial to sensory, attention, and memory processing.

Conversely, the functional connection between the hippocampus and bilateral MTG decreased after 40-Hz light exposure. Light exposure not only promotes activation of the cognitive network but also induces inhibition of the default network, causing negative activation (Karlsgodt et al., 2018; Killgore et al., 2020a). One study found that the synchronization level of the temporal lobe was affected by sensory rhythmic stimulation (Becher et al., 2015). Based on these findings, it was reasonable in the current study to use 40-Hz light to modulate the degree of synchronization and memory processing patterns between brain regions related to the processing stimuli. Stark et al. showed in their research that higher correct rates were associated with weaker activation (Hong et al., 2016b), which consumes less synchronization coupling between regions. This result shows inhibition between the hippocampus and the default mode network, which is in line with the neural efficiency hypothesis (Neubauer and Fink, 2009). Light modulated at 40 Hz maintains cognitive ability and also requires fewer neural resources to be used, indicating a more efficient information processing mechanism, thus indicating that modulating the frequency of light may be an effective method to optimize neurocognitive function.

In summary, the current study aimed to examine the effect of 40 and 0-Hz light exposure on the regulation of brain activity patterns. The results show that the hippocampus and PFC had different response characteristics to different frequencies of light. Only 40-Hz light evoked enhanced activity in the hippocampus and caused changes in functional connection with other brain regions. Such evidence of distinct patterns of activity for 40 and 0-Hz stimuli suggests that the brain regions distributed in memory-related brain network have different patterns of response to frequency modulation. These findings are consistent with the notion that mnemonic discrimination is limited to the hippocampus, and further predict the optimization effect of gamma light stimulation on brain function related to memory function. The data suggest that the use of 40-Hz frequency light can indeed change the hippocampal activity and brain function connections related to memory tasks, as we expected. With due consideration to the limitations caused by the lack of robustness in changes in cognitive performance, we posit that the results of this study may provide clues for the establishment of a systematic light stimulus-brain response correspondence.

Several limitations should be considered. First, the sample size was small in the present study, which may have limited the statistical power and resulted in overestimates and low reproducibility. Consequently, further studies with larger sample sizes are needed to further characterize the neural mechanisms underlying light exposure. Moreover, the sample characteristics were somewhat heterogeneous. Considering that differences in individual responses are a hot issue in neuromodulation research, the difference observed in the results may also be a normal or universal phenomenon. However, at least in the analysis performed in this study, strict corrections were made to ensure the reliability of the various activation and connection results displayed by the group level. Participants performed fMRI experiments as quickly as possible within 10 min of the end of the light intervention. Although studies have shown that the effect of light exposure lasts for more than 30 min, the peak time of the effect induced by 40-Hz light in the current study still needs to be explored in future research. Additionally, the single trial of light intervention used in this study needs further confirmation of whether the regulation mode will change after multiple days of continuous light exposure. As only one modulation pattern (40 Hz) was used, and it was compared to no frequency modulation at all, we must ask whether memory-related brain networks or even task-/function-related brain networks have distinct preferences for frequency modulation. This may require comparison to other frequency modulations in future studies. Finally, with respect to memory task-related regulation, whether 40-Hz illumination is the optimal gamma frequency band is still a question that needs to be studied systematically.

## Data Availability Statement

The raw data supporting the conclusions of this article will be made available by the authors, without undue reservation.

## Ethics Statement

The studies involving human participants were reviewed and approved by Laboratory Ethics Committee of Shenzhen University. The patients/participants provided their written informed consent to participate in this study. Written informed consent was obtained from the individual(s) for the publication of any potentially identifiable images or data included in this article.

## Author Contributions

ZL, YaY, and JM designed experiments and wrote the manuscript. ZL, GH, YoY, FZ, ZZ, LL, and LZ conducted experiments. ZL, LL, and JM analyzed the data. All authors approved the final manuscript.

## Conflict of Interest

The authors declare that the research was conducted in the absence of any commercial or financial relationships that could be construed as a potential conflict of interest.

## Publisher’s Note

All claims expressed in this article are solely those of the authors and do not necessarily represent those of their affiliated organizations, or those of the publisher, the editors and the reviewers. Any product that may be evaluated in this article, or claim that may be made by its manufacturer, is not guaranteed or endorsed by the publisher.
